# Introduction to the Special Issue: Advanced Strategies to Preserve Quality and Extend Shelf Life of Foods

**DOI:** 10.3390/foods11071052

**Published:** 2022-04-06

**Authors:** Amalia Conte, Matteo A. Del Nobile

**Affiliations:** Department of Agricultural Sciences, Food and Environment, University of Foggia, Via Napoli 25, 71122 Foggia, Italy

We are pleased to present this Special Issue, which includes 13 papers that highlight the most important research activities in the field of food quality assurance and shelf-life extension. The goal of this Special Issue was to broaden the current knowledge of advanced approaches to guarantee the maintenance of the properties of packaged products during storage. The most consolidated strategies in the literature concern the use of heat and modified atmospheres. However, knowledge gained in the sector has broadened the perspective and found valid and effective alternative in the use of bioactive compounds, industrial food by-products, adoption of active packaging solutions or the application of novel mild treatments, such as pulsed light, ultrasounds, high-pressure processing and cold plasma.

The 11 research articles/communication/ and 2 reviews that comprise this Special Issue highlight the most recent research and investigations into this exciting area, covering the following topics: (i) vacuum packaging; (ii) cork closures; (iii) innovative active packaging; (iv) emerging technologies; (v) the reuse of by-products; and (vi) secondary shelf life.

Interesting results that bring to light the issues concerning the effects of vacuum packaging on surface color and lipid oxidation of beef steaks were presented by Reyes et al. [1]. The results from this study suggest that the use of vacuum packaging for beef steaks is plausible for maintaining quality characteristics during extended display periods.

The study of Amaro et al. [2] aimed at investigating the impact of different technical cork stoppers on the quality preservation and shelf life of sparkling wines. The volatile compositions of two Italian sparkling wines sealed with a sparkling cork with two natural cork discs (2D) and a micro-agglomerated (MA) cork were determined during bottle aging (12 to 42 months) after disgorging. The results unveiled that the type of closure has a greater impact on the volatile composition of sparkling wines at longer post-bottling periods, and 2D stoppers preserve the fruity and sweet aromas of sparkling wines better after 42 months of bottle storage.

The next four papers dealt specifically with the effects of active packaging on food shelf life. In particular, Ambrosio et al. [3] proved the positive effects of an active polypropylene-based packaging functionalized with the antimicrobial peptide 1018K6 on microbial growth, physicochemical properties and sensory attributes of raw salmon fillets and hamburgers of Sarda sarda during storage. Roy et al. [4] developed a pullulan/chitosan-based multifunctional edible composite film by reinforcing mushroom-mediated zinc oxide nanoparticles (ZnONPs) and propolis. The system was advantageously used for wrapping pork belly. Gutiérrez-Jara et al. [5] coated sweet cherries by electro-spraying with an edible nano-emulsion of alginate and soybean oil, with or without a CaCl_2_ cross-linker to reduce fruit cracking. It was interesting to observe that the use of the nano-emulsion + CaCl_2_ coating on sweet cherries helps to reduce cracking and maintain fruit quality at 4 °C for about 1 month. Finally, Socaciu et al. [6] studied the effects of a whey-protein-isolate-based film incorporated with tarragon essential oil on the quality and shelf-life of refrigerated brook trout. The selected essential oil conferred antioxidant and antimicrobial properties to the film. Thus, the developed active packaging system could be a promising material for fresh fish packaging.

As regards the adoption of active compounds of natural origin, two papers have been published, one dealing with shelf-life extension of chilled pork by optimal ultrasonicated ceylon spinach (*Basella alba*) extracts [7] and another one on the potential of algae extracts for extending the shelf life of rainbow trout (*Oncorhynchus mykiss*) fillets [8]. In the first study, Phimolsiripol et al. [7] found that fresh pork treated with the ultrasonicated extracts at 100 and 120 mg/mL had lower values of thiobarbituric acid reactive substances (TBARS) than the control (without dipping). From the food safety standpoint, as measured by the total microbial count, the fresh pork dipped with 100–120 mg/mL spinach extract could be kept at 0 °C for 7 days, 2 to 3 days longer than control meat at 0 and 4 °C, respectively. The results of Saez et al. [8] on the shelf life of rainbow trout demonstrated that algae extracts are also naturally effective agents for preserving fish.

In the context of natural compounds used for shelf-life extension, another two studies have been also published in the current Special Issue. This is the case of one article and one review dealing with fruit and vegetable by-products, whose valorization is considered a hot topic. In the article of Panza et al. [9], olive paste, a by-product from olive oil production, was valorized as breading for fresh fish sticks stored for 15 days at 4 °C. The results proved that the enrichment with olive paste increased the total phenols, the flavonoids and the antioxidant activity of the breaded fish samples compared to the control, without compromising the sensory parameters. The overview of Nardella et al. [10] collects the recent applications of fruit and vegetable by-products as valid components to prolong food shelf life. This review provides a detailed picture of the state-of-art of the literature on the topic in the last 10 years. The review highlights the potential of by-products and the clear advances in terms of food sustainability, even though the current situation still limits by-product diffusion. The authors also underlined that for future perspectives of by-products recycling, multidisciplinary research is of striking importance, as it is able to promote the scale-up of by-products and encourage their adoption at the industrial level.

As regards the emerging technologies and food shelf life, one article and one review were published in the current Special Issue. In particular, the article deals with the effects of gaseous ozone on microbiological quality of Andean blackberries (*Rubus glaucus* benth) [11]. Andean blackberries are highly perishable. Ozone was applied prior to storage at 0.4, 0.5, 0.6 and 0.7 ppm for 3 min, and this treatment was found effective in maintaining the quality of blackberries throughout refrigerated storage. The authors suggest that higher doses could be advisable to enhance its antimicrobial activity. The review of Tavares et al. [12] deals with emergent preservation techniques (chilling and super-chilling) as a complement, or even replacement of conventional preservation methodologies (refrigeration and freezing), to assure fish safety and extend shelf life without compromising food safety. In addition, the use of novel food packaging methodologies (edible films and coatings) was also presented and discussed, along with a new storage methodology, hyperbaric storage, that uses storage pressure control as a hurdle microbial development and slows down organoleptic decay at subzero, refrigeration and room temperatures.

One paper dealing with secondary shelf life (SSL) is also included in this Special Issue. SSL represents the time after package opening during which the food product retains a required level of quality. The study of Nicosia et al. [13] suggests the possibility to significantly extend or even omit the SSL indications for industrial pesto sauces because the product remained acceptable for a time longer than that reported on the label. This study could have practical outcomes at the domestic level in terms of food waste reduction and at industrial level in terms of reduced household stock turnover and consequent cost savings.

Taken together, these studies are clear evidence of how the achievement of food shelf-life extension is still a complex and multifaceted process. Food manufacturers have to meet consumer demands for freshness and convenience without compromising the safety of foods, and the food industry is thus continuously challenged and seeking sustainable and practical methods to ensure the safety of products and guarantee the maximum level of security for consumers. The innovative and exciting research included in this Special Issue highlights the interest and potential of this emerging area, addressing some of the most pressing global issues.

In summary, all the papers published in this Special Issue highlighted a large portion of the research activities in the field of advanced application of novel processing, antimicrobial/antioxidant substances as well as from by-products and active packaging. The development of these topics and the exploration of their combined use will remain a very active research area in the coming decades.

We sincerely hope that the readers will find this Special Issue interesting and informative.
